# Efficacy and Side Effects of Narrowband-UVB in Early Stage Cutaneous T-Cell Lymphoma in Jordanian Patients

**DOI:** 10.1155/2014/951821

**Published:** 2014-02-19

**Authors:** Salah A. Abdallat, Ayman S. Alqaqaa, Nidal A. Obaidat, Rameh F. Alnueimi

**Affiliations:** Department of Dermatology, King Hussein Medical Center, Amman 11941, Jordan

## Abstract

*Background*. Many studies, on light-skinned patients, suggested narrowband-UVB to be effective and safe for the treatment of early stage cutaneous T-cell lymphoma. *Objectives*. To evaluate the efficacy and side effects of narrowband-UVB in treatment of early stage cutaneous T-cell lymphoma in patients with skin phototypes III, IV, and V. *Methods*. A total of 27 patients with the diagnosis of early stage cutaneous T-cell lymphoma were involved in this prospective study. All patients received narrowband-UVB as monotherapy until clearance or a maximum of 42 sessions. Patients with complete clearance were followed for six months or relapse. Rate of clearance, number of sessions, and cumulative narrowband-UVB dose needed to achieve clearance, percentage of patients remaining in remission at 6 months, and side effects were analyzed. *Results*. Within 5–14 weeks (15–42 sessions), using cumulative narrowband-UVB dose ranging from 17.3 to 48.2 J/cm^2^, complete remission was achieved in 76.4% of patients. The rest of the patients achieved partial remission. Six months after discontinuation of the treatment, 42.8% of patients with complete remission remained in remission. Transient erythema in 11.1% of patients and mild hyperpigmentation in 14.8% of patients were the only side effects encountered during this study. *Conclusion*. We conclude that narrowband-UVB phototherapy is safe and effective for the treatment of early stage cutaneous T-cell lymphoma in darker-skinned patients.

## 1. Introduction 

Cutaneous T-cell lymphoma (CTCL) is a group of lymphoproliferative disorders with clonal expansion of T helper cells, or rarely T suppressor/killer cells or NK cells, with localization to the skin. This group is characterized by an increased CD4+ cells: CD4/CD8 > 10, and/or an expansion of T cells with a loss of 1 or more of the normal T-cell antigens (CD2, CD3, and CD5) [1].

Cutaneous T-cell lymphomas are very rare, with a prevalence of 5/1000000 per year [1, 2]. They are classified into a group with indolent clinical behavior, which includes mycosis fungoides (MF) and its variants (62%), and primary cutaneous CD30+ lymphoproliferative disorders (26%) and a group with aggressive clinical behavior (12%) like Sézary syndrome and adult T-cell leukemia/lymphoma [2].

MF, the commonest form of CTCL, presents as patches and plaques over trunk and proximal extremities, without internal involvement. It has a predilection for older adults and male gender [3–5]. MF is classified into early (stage IA, IB, and IIA) and advanced (stage IIB, III and IV) stages [2, 3].

In early stages, the clinical and pathological presentation are similar to other inflammatory dermatoses, for example, Atopic eczema. Repeated skin biopsies are usually needed to establish the diagnosis [4, 5]. Treatment of early stage MF includes topical agents (corticosteroid, nitrogen mustards, carmustine, and bexarotene) [6], electron beam therapy, and phototherapy including ultraviolet light, excimer laser, and photodynamic therapy [7–9].

Many studies involving Western populations showed that NB-UVB is effective for early stage MF [7, 8], but there is lack of studies on NB-UVB in early MF in darker-skinned patients. In this study, we analyzed the efficacy and safety of NB-UVB in patients with skin phototypes III, IV, and V having early stage MF.

## 2. Methods 

This prospective study was done at the Department of Dermatology, King Hussein Medical Center, Amman, Jordan, between October 2010 and July 2012. After obtaining ethical clearance and providing informed consent, 27 adult patients with clinical and pathological diagnosis of CTCL were enrolled in this study. Patients with photosensitivity diseases or photosensitizing medications were excluded.

Staging was done based on type of skin lesion (patch, plaque, and tumor), percentage of body surface area involved, and involvement of lymph nodes, peripheral blood, and metastasis as assessed by physical examination, complete blood count and chemistry, blood morphology, U/S, and CT radiologic study [2, 3]. According to the TNM staging of the World Health Organization-European Organization for Research and Treatment of Cancer (WHO-EORTC) [2], 8 patients were classified as stage IA, and 19 patients were classified as stage IB.

All patients were treated with NB-UVB phototherapy as monotherapy. They were started at 70% of minimal erythema dose (MED), with 20% increase per session, three times weekly, until clearance or a maximum of 42 sessions.

At each visit, responses to phototherapy were independently assessed by four dermatologists based on clinical reduction or clearance of skin lesions, using standardized photographs. Skin biopsy was not repeated after treatment.

Response was considered complete if clearance is >95%, partial if clearance is 50–95%, or non if clearance is <50%. Adverse effects were assessed as well.

Patients with complete clearance were followed for six months or relapse.

Rate of clearance, number of sessions and cumulative NB-UVB dose needed to achieve clearance, percentage of patients remaining in remission at 6 months, and percentage of side effects were investigated.

## 3. Results

Patients' ages ranged from 21 to 63 years, with a mean of 46.3 years. Males accounted for 59% of the patients. All patients included in this study were skin phototypes III, IV, and V, as 16 patients (59%) of them were skin phototype III, 7 patients (26%) were phototype IV, and 4 patients (15%) were phototype V.

Complete remission was achieved in 76.4% (21/27) of patients, within 5–14 weeks (mean 12.7 weeks). During this period, 15–42 sessions (mean 28.9 sessions), equivalent to a cumulative NB-UVB dose of 17.3–48.2 J/cm^2^ (mean 38.7 J/cm^2^), were needed to achieve this rate of complete remission. In 23.6% of our patients (6/27), NB-UVB monotherapy was continued for 14 weeks (42 sessions, 45.7–48.2 J/cm^2^, mean 46.6 J/cm^2^) with the result of partial remission (64%–82% clearance of skin lesions). See Table 1.

During the six-month follow up period, 42.8% of the 21 patients with complete remission (9/21) remained in remission. Rates of complete remission, number of sessions to complete clearance, and NB-UVB dose in the three skin phototypes (III, IV, and V) are shown in Table 2. The statistical significance of the differences between the three groups in rates of complete remission, number of sessions, and NB-UVB dose was measured using *P* value as seen in Table 3.

Side effects were limited to 3 cases of transient erythema (11.1%) and 4 cases of mild hyperpigmentation (14.8%).

## 4. Discussion

The standard staging system for mycosis fungoides is based upon the evaluation of the skin (T), lymph nodes (N), visceral involvement (M), and blood (B) according to the WHO-EORTC TNM staging of MF [2].

Cases with early stage MF (stages I-IIA) usually have benign, chronic, and recurrent course but rarely progress to more aggressive forms. So, early and appropriate treatment is crucial to ascertain good prognosis [6].

In most patients with early stage MF, the disease remains confined to the skin for years and decades, making skin-directed therapy the optimal treatment [6]. Topical emollients, corticosteroids, or topical bexarotene are considered first line treatments for limited patches or thin plaques. Total skin electron beam radiation is the best used for generalized thick plaques in early stages of MF [7, 8].

Phototherapy is useful in the treatment of all stages in early MF. This includes the use of PUVA, NB-UVB, broadband UVB (BB-UVB), UVA-1, photodynamic therapy, and excimer laser [7, 8]. Of these, PUVA and NBUVB have been used most commonly [7, 10]. PUVA treatment is considered by many as the standard therapy for the early stages of MF. However, many comparative studies have shown NBUVB and PUVA to achieve comparable rates of complete remission and maintenance of remission. NBUVB has the added advantage of a lower incidence of adverse events [7, 10].

However, most studies conducted on the use of NB-UVB for the treatment of early stage CTCL were conducted in Caucasians, but not in darker-skinned patients. While some authors believe that clinical stage of CTCL is more important than skin phototype in determining the response to NB-UVB, others have doubts about the effectiveness of NB-UVB in dark-skinned patients because of the photoprotective function of melanin [10].

In comparison with similar studies, patients enrolled in our study were 27, compared to a range from 6 patients in the study by Hofer et al. [11] to 68 patients in the study by Pavlotsky et al. [12]. The small number of patients in these studies is due to the rare nature of the disease.

In the current study, out of 27 patients, complete remission was achieved in 21 patients (76.4%), within 5–14 weeks (mean 12.7 weeks). A total of 15–42 sessions (mean 28.9 sessions), equivalent to a cumulative NB-UVB dose of 17.3–48.2 J/cm^2^ (mean 38.7 J/cm^2^), were needed to achieve this rate of complete remission. In the literature concerning treatment of early stage disease by NB-UVB, the rate of complete remission ranged from 54% by Gathers et al. [13] to 91% by Gökdemir et al. [14].

Assessment of posttreatment remission was based on clinical clearance of skin lesions, as many authors believe that clinical remission correlates well with histopathological improvement [15]. Based on this, we did not perform a skin biopsy to assess response to treatment.

Rates of complete remission were comparable in the different skin phototypes (81.2%, 71.4%, and 75% in skin types III, IV, and V, resp.), as evidenced by *P* values comparing skin type III with IV, IV with V, and III with V, which were all statistically insignificant (*P* value > 0.01). The differences in number of sessions between skin types III and IV and between IV and V were statistically insignificant (*P* value > 0.01), unlike the significant difference between III and V (*P* value < 0.01). The differences in NB-UVB dose were insignificant between skin types III and IV and between IV and V (*P* value > 0.01), but significant when comparing skin type III with V (*P* value < 0.01). These results are summarized in Tables 2 and 3.

Compared to light-skinned patients, these results suggest that NB-UVB is almost equally effective in treating early stage CTCL in dark-skinned patients, but more treatment sessions and higher cumulative NB-UVB doses are needed, especially in skin type V.

During the six-month follow-up period, 42.8% of our 21 patients with complete remission (9/21) remained in remission. In similar studies, follow-up was continued until relapse, reporting a relapse-free duration of 4.5 months by Ghodsi et al. [15] to 26 months by Boztepe et al. [16].

In 23.6% of our patients, partial remission was achieved. This falls within the range of partial remission rates reported in literature, from 6% in the study of Pavlotsky et al. [12] to 35% by Coronel-Pérez et al. [17].

In our group of 27 patients, treatment with NB-UVB was well tolerated and side effects were limited to 3 cases of transient erythema (11.1%) and 4 cases of mild hyperpigmentation (14.8%).

In conclusion, we found that NB-UVB is safe and effective in treatment of early stage CTCL, but more sessions and higher NB-UVB doses are needed especially in skin type V. More investigative studies, using larger populations, are needed to attain the optimal goal of longer lasting remission rates.

## Figures and Tables

**Table 1:** Rates of early stage CTCL response to NB-UVB phototherapy in relation to duration and dose of NB-UVB.

	Complete remission	Partial remission
Patients: % (number)	76.4% (21/27)	23.6% (6/27)
Duration of treatment in weeks: range (mean)	5–14 (12.7)	14
No. of sessions: range (mean)	15–42 (28.9)	42
Cumulative NB-UVB dose in J/cm^2^: range (mean)	17.3–48.2 (38.7)	45.7–48.2 (46.6)
Maintenance of remission: % (number)	42.8% (9/21)	N.A.

**Table 2:** Rates of complete remission, number of treatment sessions, and NB-UVB dose in relation to skin phototype.

	Skin type III	Skin type IV	Skin type V
Complete remission: % (number)	81.2 (13/16)	71.4 (5/7)	75 (3/4)
Number of sessions: range (mean)	15–34 (24.7)	16–39 (32.2)	19–42 (36.7)
NB-UVB dose in J/cm^2^: range (mean)	17.2–36.3 (37.1)	19.7–42.1 (39.1)	20.9–48.2 (44.3)

**Table 3:** *P* values comparing rates of complete remission, number of treatment sessions, and NB-UVB dose between skin types III, IV, and V.

	Rates of complete remission	Number of sessions	NB-UVB dose in J/cm^2^
*P* value (skin types III and IV)	0.031	0.027	0.043
*P* value (skin types IV and V)	0.043	0.037	0.037
*P* value (skin types III and V)	0.041	0.0073	0.0086
